# Prophylactic Antibiotics Before Insertion of Tunneled Hemodialysis Catheters: A Nationwide Cohort Study

**DOI:** 10.1016/j.xkme.2025.101042

**Published:** 2025-06-02

**Authors:** Benjamin Lazarus, Sradha Kotwal, Martin Gallagher, Kathryn Higgins, Sarah Coggan, Nicholas A. Gray, Girish Talaulikar, Kevan R. Polkinghorne, REDUCCTION investigators

**Affiliations:** 1 The George Institute for Global Health, University of New South Wales, Sydney Australia; 2 Department of Medicine, Monash University, Clayton, Victoria, Australia; 3 Department of Nephrology, Monash University, Clayton, Victoria, Australia; 4 Centre for Health Services Research, University of Queensland, Woolloongabba, Queensland, Australia; 5 Prince of Wales Hospital, University of New South Wales, Sydney, Australia; 6 South Western Sydney Campus, University of New South Wales, Sydney Australia; 7 Sunshine Coast University Hospital, Birtinya, Queensland Australia; 8 University of the Sunshine Coast, Sippy Downs, Queensland Australia; 9 Department of Nephrology, The Canberra Hospital, Garran, ACT, Australia; 10 Australian National University School of Medicine, Acton, ACT, Australia; 11 Department of Epidemiology and Preventive Medicine, Monash University, Prahran, Victoria, Australia

**Keywords:** Antimicrobial, prevent, CVC, bloodstream, infection, renal failure

## Abstract

**Rationale & Objective:**

It is unknown whether administration of prophylactic systemic antibiotics immediately before tunneled catheter insertion can prevent early hemodialysis catheter-related bloodstream infections (HDCRBSI). We aimed to estimate the effect of systemic prophylactic antibiotics on early HDCRBSI.

**Study Design:**

An observational secondary analysis using data from the nationwide REDUcing the burden of dialysis Catheter ComplicaTIOns: a National approach trial.

**Setting & Participants:**

Adults with an incident hemodialysis catheter inserted in one of 37 Australian nephrology services from December 2016 to March 2020.

**Exposure:**

Service-wide policy of systemic prophylactic antibiotic use before tunneled catheter insertion determined by response to a prestudy survey.

**Outcome:**

HDCRBSI within 14 days of catheter insertion, independently adjudicated by a blinded panel using modified Infectious Diseases Society of America criteria.

**Analytical Approach:**

Multilevel logistic regression to compare outcomes among antibiotic-using and nonusing services.

**Results:**

Six services (900 patients) used prophylactic antibiotics, and 23 services (3,702 patients) did not. Among the 1,196 tunneled catheters that were inserted in antibiotic-using services, 4 (0.3%) had HDCRBSI and another 10 (0.8%) had infectious removal within 14 days of insertion. Among the 5,027 tunneled catheters inserted in nonantibiotic-using services, 40 (0.8%) had HDCRBSI and another 41 (0.8%) had infectious removal within 14 days. The odds of early HDCRBSI were not significantly different between antibiotic-using and nonusing services in the unadjusted (OR, 0.42; 95% CI, 0.15-1.17) or adjusted models (adjusted OR, 0.59; 95% CI, 0.20-1.80).

**Limitations:**

Prophylactic systemic antibiotic use was determined at a service level and was not randomly assigned to individuals.

**Conclusions:**

In Australia, less than 1% of tunneled catheters had confirmed HDCRBSI within 14 days of insertion. Routine administration of prophylactic antibiotics before insertion of tunneled cuffed catheters was not associated with a reduced occurrence of early HDCRBSI within 14 days.

## Plain-Language Summary

Millions of people worldwide with kidney failure rely on a central venous catheter to facilitate life-sustaining hemodialysis treatments. Unfortunately, catheters can lead to dangerous bloodstream infections, particularly during the first 2-weeks after insertion. It is unknown whether administration of prophylactic antibiotics before catheter insertion can prevent such infections, and real-world practice is mixed. We conducted a post-hoc analysis of the REDUcing the burden of dialysis Catheter ComplicaTIOns: a National approach trial and found that there was no statistically significant difference in the proportion of catheters that were affected by early bloodstream infection in prophylactic-antibiotic-using services (0.3% of 1,196 catheters) compared with nonusing services (0.8% of 5,027 catheters). More observational studies are warranted pending large, randomized trials.

Tunneled central venous catheters (CVCs) are commonly used for hemodialysis (HD) vascular access^1^ but are a source of burdensome and costly health care-associated infections,^2^^,^^3^ particularly during the first month after catheter insertion.^4^ Despite these risks, 80% of patients commence long-term HD using a CVC in the United States,^5^ rather than arteriovenous access,^6^ and up to 9% of patients using a long-term HD catheter are affected by HD catheter-related bloodstream infection (HDCRBSI) in the first year.^7^ The risk of HDCRBSI is highest in the early period after insertion, probably because the insertion procedure provides more opportunity for contamination, and the fibrous cuff, which may be a barrier to extra-luminal transmission of microorganisms, takes time to form.^8^

It is unknown whether prophylactic systemic antibiotics immediately before tunneled HD catheter insertion can prevent early HDCRBSI. The 2022 International Society for Peritoneal Dialysis (PD) guidelines recommend systemic prophylactic antibiotics immediately before PD catheter placement,^9^ to prevent PD peritonitis within the first 2 weeks after catheter insertion,^10^ and it is plausible that the same principle may apply to insertion of tunneled CVCs. However, the 2011 Centers for Disease Control and Prevention guidelines recommend against prophylactic antibiotics before tunneled CVC insertion,^11^ based on studies of CVCs for oncology patients,^12^ including fully implantable CVCs,^13^^,^^14^ which may not be generalizable to the modern HD setting. HD vascular access guidelines have not addressed the question of prophylactic antibiotics for tunneled catheters,^15^^,^^16^ and there is wide variation in the use of prophylactic antibiotics for tunneled HD catheters within Australia and internationally.^17^^,^^18^ Given that tunneled CVCs are widely used globally, quantifying the risks and benefits of systemic prophylactic antibiotics is essential to inform shared decision-making by patients and clinicians.

In Australia, a nationwide survey of catheter practices and a subsequent trial assessing HD catheter complications were recently completed.^18^^,^^19^ Using these data, we aimed to estimate the effect of a service-wide policy of systemic prophylactic antibiotics on early HD catheter infections. We hypothesized that HD catheters that were inserted in services that had a policy of administering systemic prophylactic antibiotics immediately before insertion had a lower risk of early HDCRBSI or catheter infection requiring removal.

## Methods

### Study Design and Population

The design of the REDUcing the burden of dialysis Catheter complications: a national approach (REDUCCTION) trial and preceding survey have been described previously.^18^, ^19^, ^20^ REDUCCTION was a stepped-wedge cluster randomized trial that enrolled adults who received HD by an incident CVC across 37 Australian nephrology services from December 20, 2016 to March 31, 2020. The trial assessed a suite of practices designed to prevent HDCRBSI but did not make any recommendations for services to modify their existing practice with respect to the use of prophylactic systemic antibiotics before CVC insertion. In this post-hoc analysis, nontunneled HD catheters and any HD catheters with an unknown date of insertion were excluded. This trial adhered to the Declaration of Helsinki, and ethical approval was obtained across all relevant jurisdictions. The trial was registered in the Australia and New Zealand clinical trials registry on June 23, 2016 (ACTRN12616000830493).

### Exposure

All nephrology services that were scheduled to commence the REDUCCTION trial were invited to participate in a pretrial survey and were sent a follow-up reminder to complete the survey if no response was received. From May 2016 to October 2016, responses were received from 31 of the 37 services that subsequently commenced trial data collection from December 2016. In the prestudy survey, the vascular access leader at each site was asked, “Do you give prophylactic antibiotics at the time of insertion of tunneled catheters” with 3 response options: “No,” “Unsure,” or “Yes.” The follow-up question for a “Yes” answer was, “What prophylactic antibiotic(s) is the first choice at the time of insertion of tunneled catheters? Tick all that apply” with response options: “First-generation cephalosporin (eg, cephalothin and cephazolin),” “Vancomycin,” “Gentamicin,” “Unsure,” and “Other (please specify).” Catheters that were inserted in services that reported in the survey to routinely using prophylactic antibiotics before HD-CVC insertion were assumed to be exposed to prophylactic antibiotics, whereas catheters inserted in services that did not routinely use prophylactic antibiotics were considered unexposed. Catheters inserted in services that reported being unsure of whether they used prophylactic antibiotics were excluded.

### Outcomes

The primary outcome of this study was HDCRBSI within the first 14 days after insertion, in which HDCRBSI cases were centrally adjudicated by a blinded panel and defined using a modified Infectious Diseases Society of America definition.^20^ A time frame of 14 days was chosen because it has been used for one trial assessing prophylactic antibiotics in patients with PD^10^ and was a biologically plausible time frame within which prophylactic antibiotics might prevent infection.

Interruptions to catheter function are a key priority for patients,^21^ and therefore infectious catheter removal, as assessed by the treating nephrologist, or confirmed HDCRBSI within the first 14 days was used as a composite secondary endpoint. We also assessed confirmed HDCRBSI within 30 days as an additional secondary outcome measure, because some trials from the PD literature suggested that infections within this time frame may also be related to contamination and colonization at the time of insertion.^22^

### Study Measures

Service characteristics included Australian state or territory, urban or regional location, and size based on the total number of patients requiring long-term HD in the entire service on the December 31, 2016. Large services were defined as those that treated more than 400 patients receiving long-term HD in December 2016. Participant age, gender, comorbid diabetes mellitus, and use of immunosuppressant medication were recorded at baseline. The multifaceted intervention implemented during the original trial was documented and considered as a covariate, absent for catheters inserted during the baseline phase and present among catheters inserted during the intervention phase in each service.

### Statistical Methods

Baseline characteristics of services, participants, and catheters are presented as number (percentages), means and medians.

The raw service-wide proportions of tunneled catheters that experienced HDCRBSI within the first 14 days were compared between services that used and did not use prophylactic antibiotics. The crude odds of HDCRBSI were compared between catheters in antibiotic-using services and nonusing services by logistic regression without adjustment. Adjusted odds were calculated with multilevel logistic regression, with random effects for service and patients within service and fixed effects for whether the catheter was inserted during the intervention phase of the original trial, service size (<200, 200-400, or >400 patients receiving HD), region (metro vs regional), categorical patient age (<50, 50-60, 60-70, 70-80, or >80 years), gender, diabetes mellitus, immunosuppressant use, previous catheter use, proceduralist, and site of catheter insertion.

Two sensitivity analyses were performed. First, we restricted catheters to those inserted before April 1, 2018, before the intervention was implemented in any site. Second, we excluded tunneled catheters that were inserted for acute kidney injury (AKI) because a proportion of patients with AKI may have already been receiving antimicrobials for sepsis, which precipitated AKI. The α level was 0.05. Statistical software was used for all analyses (Stata/BE, version 18.0).

## Results

**Figure 1:**
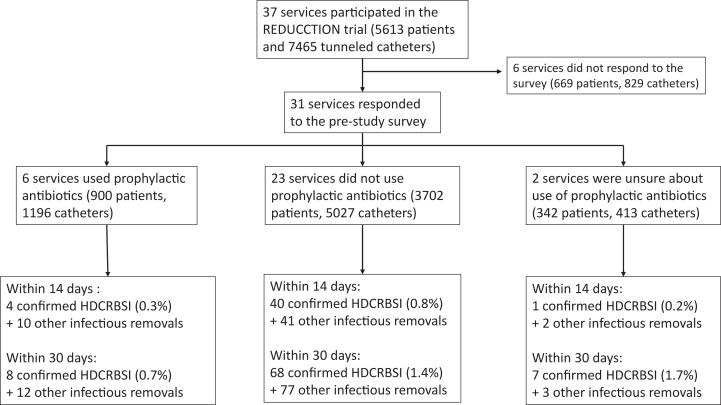
Flowchart of participating services and the number of confirmed HDCRBSI and other infectious catheter removals within the first 14 and 30 days. HDCRBSI, hemodialysis catheter-related bloodstream infections.

Of the 31 Australian nephrology services that responded to the prestudy survey and subsequently participated in the REDUCCTION trial, 6 reported the use of prophylactic systemic antibiotics immediately before tunneled catheter insertion, 23 reported not using prophylactic antibiotics, and 2 services reported being unsure about whether prophylactic antibiotics were used (Fig 1). All 6 services that used prophylactic antibiotics reported the use of first-generation cephalosporins.

Baseline characteristics of services, patients, and tunneled catheters are presented in Table 1. The 6 services that used prophylactic antibiotics were in metropolitan and regional locations across the country. Out of 3 large services, 2 reported prophylactic antibiotic use. In total, 4,603 patients and 6,223 tunneled catheters were analyzed. The mean patient age was 60 years, 40% were female, 64% were White, 43% had diabetes mellitus, and 12% were taking immunosuppressant medication. A larger proportion of patients in antibiotic-using services was immunosuppressed. Catheters inserted in services that used prophylactic antibiotics were more frequently inserted by surgeons and sited in the right internal jugular vein (Table 1).

**Table 1:** Baseline Characteristics of Services That Used and did not Use Prophylactic Antibiotics, and Patients and Tunneled Catheters in These Services

	Prophylactic Antibiotic	No Antibiotic	Total
**Service characteristics**			
Number of services	6	23	29
Satellite units, mean ± SD	6.5 ± 9.2	3.8 ± 3.4	4.4 ± 5.0
State or territory, n (%)			
NSW/ACT	3 (50.0)	8 (34.8)	11 (37.9)
VIC/TAS	2 (33.3)	4 (17.4)	6 (20.7)
QLD/NT	1 (16.7)	8 (34.8)	9 (31.0)
SA/WA	0 (0)	3 (13.0)	3 (10.3)
Service size, n (%)			
Small (<200 patients receiving HD)	3 (50.0)	9 (39.1)	12 (41.4)
Medium (200-400 patients receiving HD)	1 (16.7)	13 (56.5)	14 (48.3)
Large (>400 patients receiving HD)	2 (33.3)	1 (4.3)	3 (10.3)
Regional location, n (%)	2 (33.3)	4 (17.4)	6 (20.7)
**Patient characteristics**			
Number of patients	900	3,703	4,603
Age (y), mean ± SD	60.7 ± 16.0	60.2 ± 15.9	60.3 ± 15.9
Female, n (%)	352 (39.1)	1,466 (39.6)	1,818 (39.5)
Ethnicity, n (%)			
Asian	40 (4.4)	290 (7.8)	330 (7.2)
First Nations	37 (4.1)	474 (12.8)	511 (11.1)
White	481 (53.4)	2,464 (66.5)	2,945 (64.0)
Other or not reported	342 (38.0)	475 (12.8)	817 (17.7)
Diabetes mellitus, n (%)	406 (45.1)	1,590 (42.9)	1,996 (43.4)
Immunosuppressed, n (%)	152 (16.9)	439 (11.9)	591 (12.8)
**Catheter characteristics**			
Number of tunneled catheters	1,196	5,027	6,223
Patient catheter number, n (%)			
1st	900 (75.3)	3,703 (73.7)	4,603 (74.0)
2nd	175 (14.6)	793 (15.8)	968 (15.6)
≥3rd	121 (10.1)	531 (10.6)	652 (10.5)
Indication for catheter, n (%)			
Maintenance dialysis	488 (40.8)	1,874 (37.3)	2,362 (38.0)
AKI	260 (21.7)	1,550 (30.8)	1,810 (29.1)
Transfer from PD	160 (13.4)	607 (12.1)	767 (12.3)
AVF/AVG complication	271 (22.7)	916 (18.2)	1,187 (19.1)
Other	17 (1.4)	80 (1.6)	97 (1.6)
Right internal jugular site, n (%)	985 (82.4)	3,825 (76.1)	4,810 (77.3)
Proceduralist, n (%)			
Radiology	512 (42.8)	4,106 (81.7)	4,618 (74.2)
Surgery	490 (41.0)	154 (3.1)	644 (10.3)
Nephrology	133 (11.1)	548 (10.9)	681 (10.9)
Other	61 (5.1)	219 (4.4)	280 (4.5)

Abbreviations: ACT, Australian Capital Territory; AKI, acute kidney injury; AVF, arteriovenous fistula; AVG, arteriovenous graft; HD, hemodialysis; NT, Northern Territory; TAS, Tasmania; VIC, Victoria; WA, Western Australia.

**Figure 2:**
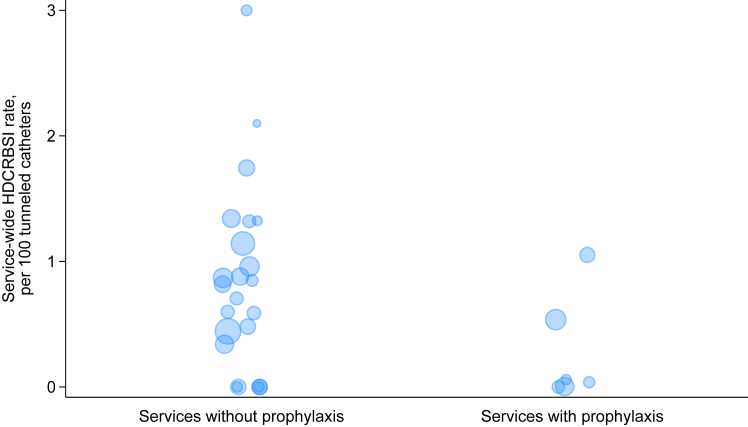
Scatter plot of the service-wide proportion of tunneled hemodialysis catheters that were complicated by HDCRBSI in services that did not use prophylactic antibiotics and those that did. The size of the marker reflects the total number of catheters at that service. HDCRBSI, hemodialysis catheter-related bloodstream infections.

In services that did not use prophylactic antibiotics, there were 40 (0.8%) confirmed HDCRBSI and 41 (0.8%) other infectious catheter removals identified within 14 days of insertion among 5,027 incident tunneled catheters, whereas in services that used prophylactic antibiotics there were 4 (0.3%) confirmed HDCRBSI and 10 (0.8%) other infectious catheter removals among 1,196 incident tunneled catheters (Fig 1). The median service-wide rate of early HDCRBSI among nonantibiotic-using services was 0.8 events per 100 tunneled catheters (IQR, 0.34-1.25), whereas 4 of the 6 antibiotic-using services did not report any early HDCRBSI (Fig 2).

The ratio of the odds of early HDCRBSI among tunneled catheters in antibiotic-using services compared with nonusing services was 0.42 (95% confidence interval [CI], 0.15-1.17; *P* = 0.10) in the unadjusted logistic model, and was 0.59 (95% CI, 0.20-1.80; *P* = 0.36) in the 3-level mixed effects model, which adjusted for measured service, patient, and catheter characteristics (Table 2). Similar results were observed for a secondary composite outcome of either HDCRBSI or infectious catheter removal within 14 days of insertion (adjusted odds ratio [OR], 0.69; 95% CI, 0.35-1.38; *P* = 0.30), or confirmed HDCRBSI within 30 days (adjusted odds ratio, 0.53; 95% CI, 0.23-1.23; *P* = 0.14; Table 3).

**Table 2:** Odds of Confirmed HDCRBSI Within 14 Days of Insertion Among Tunneled Catheters in Services That use Prophylactic Antibiotics Compared With Services That do not use Them

Model	Odds ratio	95% CI	*P*
Unadjusted	0.42	0.15-1.17	0.10
Adjusted for intervention phase of trial	0.41	0.15-1.56	0.09
Adjusted for intervention phase of trial, and service, patient, and catheter characteristics	0.60	0.20-1.79	0.36
Adjusted for intervention phase of trial, and service, patient, and catheter characteristics as fixed effects, and service and patients within service as random effects	0.59	0.20-1.80	0.36

Abbreviations: CI, confidence interval; HDCRBSI, hemodialysis catheter-related bloodstream infections.

**Table 3:** Odds of Early Tunneled HD Catheter Infection in Services That Reported Prophylactic Antibiotic use and Those That did not, Using Alternative Definitions of the Primary Outcome

Alternative Outcome	Model	Odds Ratio	95% CI	*P*
HDCRBSI or infectious tunneled catheter removal within 14 d of insertion	Unadjusted	0.72	0.41-1.28	0.27
	Adjusted	0.69	0.35-1.38	0.30
Confirmed HDCRBSI within 30 d	Unadjusted	0.49	0.24-1.02	0.06
	Adjusted	0.53	0.23-1.23	0.14

Abbreviations: HD, hemodialysis; HDCRBSI, hemodialysis catheter-related bloodstream infections.

Estimates were consistent in sensitivity analyses that restricted the study follow-up period to April 2018, before the intervention was implemented in any service (adjusted OR, 0.83; 95% CI, 0.22-3.26; *P* = 0.80) or excluded tunneled catheters inserted for AKI (adjusted OR, 0.52; 95% CI, 0.15-1.87; *P* = 0.32).

## Discussion

In this nationwide prospective cohort study of 29 Australian nephrology services, 4,603 patients, and 6,223 tunneled catheters, the cumulative proportion of tunneled catheters complicated by HDCRBSI within 14 days of insertion was 0.8% in services that did not use prophylactic antibiotics and was 0.3% in services that did. The odds of HDCRBSI within 14 days of insertion was not significantly different between catheters in antibiotic-using and nonusing services, which persisted after adjusting for measured service, patient, and catheter level differences. Similar results were obtained using a secondary outcome of HDCRBSI or infectious catheter removal within 14 days, or confirmed HDCRBSI within 30 days, and in sensitivity analyses in which catheters inserted during the study intervention phase or those inserted for AKI were excluded.

Our study substantially adds to the existing literature. Two small randomized controlled trials have sought to determine whether systemic prophylactic antibiotics before HD catheter insertion is beneficial,^23^^,^^24^ however, the quality of both trials was low, and only 167 patients were recruited in total. In one trial conducted in Turkey, 10 out of 30 patients in the control group and 6 out of 30 patients in the antibiotics group had HDCRBSI.^23^ However, the timing of infection was unclear, neither the randomization method nor the outcome definition were provided, and the statistical methods were not adequately reported.^23^ In a second trial of 107 patients who were randomized to either vancomycin 1-2 hours after insertion of a nontunneled HD catheter or no treatment, no difference in the rate of systemic catheter-related infections was reported, with 9 out of 56 patients affected in the vancomycin arm and 10 out of 51 patients affected in the no therapy arm.^24^

One observational study conducted from 2010-2013 in the United States pragmatically assessed catheter infections by contacting the dialysis nursing staff 1 week after catheter insertion (exchange or de novo) and was reported in 2 abstracts.^25^^,^^26^ The authors observed 24 catheter infections among 2,235 catheter exchanges without prophylactic antibiotics and 5 catheter infections among 2,087 catheter exchanges with prophylactic cephazolin. However, the difference was primarily driven by a higher rate in 2010 (2.39% of catheters in the no antibiotic group vs 0.54% in the prophylactic antibiotic group), which improved in subsequent years (0.47%, 0.69%, and 0.93% in the no antibiotic group vs 0.23%, 0.36%, and 0.16% in the prophylactic antibiotic group, in 2011, 2012, and 2013, respectively; personal communication with author, November 7, 2022). Among new catheter insertions, there were 5 events among 819 insertions without antibiotics and 2 events out of 794 insertions with prophylactic cephazolin.

The absolute rate of HDCRBSI within the first 2 weeks after catheter insertion was below 1% in our study, which is consistent with the rate of early infections reported in other recent studies. In a systematic review of randomized trials of prophylactic antibiotics in oncology patients, the 2 most recent trials analyzed a total of 512 catheters and detected only 3 infections, which was substantially less than among older trials.^12^ The low rate of infection in the modern era also suggests that any absolute benefits in infection risk with prophylactic antibiotics are likely to be small, and that randomized trials to further investigate this question would require a large sample size. However, long-term CVCs are also commonly used, so a small difference in absolute risks may still affect many people at a population level.

Vascular access guidelines have not specifically addressed the question of using prophylactic systemic antibiotics for tunneled HD catheter insertion,^15^^,^^16^ which may contribute to the observed wide variation in practice.^17^^,^^18^ This study informs patient-centered discussions regarding the risks and benefits of using prophylactic antibiotics and future research approaches. Randomized trials would likely require a very large sample size and will take some time to answer this question. In the meantime, further observational evidence of early catheter-related infection rates in the presence and absence of prophylactic antibiotics is warranted to inform this highly varied practice.

Although this study benefited from the prospective nationwide sample, broad range of services, centrally adjudicated primary outcome measure, and detailed catheter, patient, and service information, it also had some limitations. First, prophylactic antibiotic use was measured at the service level before the study started, rather than at the individual catheter level throughout the entire trial. It is likely that at least some catheters in services that used prophylactic antibiotics did not get antibiotics, and that at least some catheters in services that did not use prophylactic antibiotics did get them, which would have biased the results toward the null. Second, prophylactic antibiotics were not randomly allocated, and residual confounding was therefore possible. Although a well conducted randomized trial would address the issue of residual confounding, such a study would need a large sample size and would be costly. Third, although REDUCCTION was one of the largest HD catheter studies performed to date, the small number of confirmed HDCRBSI events within 14 days of insertion limited the statistical power of the study. Finally, international nephrology services were not included in this study. Further research to assess the risks and benefits of using preinsertion antibiotics in other environments with a higher baseline rate of infection is required.

In conclusion, the odds of early HDCRBSI in services that routinely used systemic prophylactic antibiotics at the time of tunneled HD catheter insertion were not significantly different to the odds in services that did not use prophylactic antibiotics. Given that the absolute proportion of catheters with confirmed HDCRBSI within 14 days was below 1%, randomized trials to investigate this practice would need to be very large. In the meantime, guidance on this highly variable practice will be informed by observational studies.
