# Supplementary material for: Prophylactic Antibiotics Before Insertion of Tunneled Hemodialysis Catheters: A Nationwide Cohort Study
Source: Kidney Med. 2025 Jun 2;7(8):101042. doi: 10.1016/j.xkme.2025.101042 (PMC12304927; doi:10.1016/j.xkme.2025.101042)
Supplement: Supplementary File (PDF) — Table S1. [file mmc1.pdf]

Table S1. List of REDUCCTION trial investigators and partnering organizations.

Service	First name	Last name
Alice Springs Hospital	Senthil Kumar	Balakrishnan
Alice Springs Hospital	David	Fernandes
Armadale Hospital	Hemant	Kulkarni
Armadale Hospital	Casey	Light
Armadale Hospital	Jo	Ryan
Auckland Hospital	Emma	Marsh
Auckland Hospital	David	Semple
Auckland Hospital	Jason	Wei
Alfred Health	Omar	Tombocon
Alfred Health	Rowan	Walker
Alfred Health	Scott	Wilson
Austin Health	Vilma	Lleva
Austin Health	Lucy	Mwangi
Austin Health	Peter	Mount
Austin Health	Maree	Ross-Smith
Austin Health	Marieke	Veenendaal
Barwon Health	Vicki	Smith
Barwon Health	Christine	Somerville
Cairns Hospital	Shaun	Davidson-West
Cairns Hospital	Natalie	Grainer
Cairns Hospital	Stella	Green
Cairns Hospital	Murty	Mantha
Cairns Hospital	Kati	Thiessen
Canberra Hospital	Girish	Talaulikar
Canberra Hospital	Alison	Winsbury
Canberra Hospital	Irene	Yao
Canberra Hospital	Emily	Neville
Concord Hospital	Khalilah	Marquez
Concord Hospital	Mona	Razavian
Concord Hospital	Lisa	Tienstra
Concord Hospital	Glenn	Stewart
Eastern Health	Cathy	Chan
Eastern Health	Peta	McLean
Eastern Health	Lawrence	McMahon
Eastern Health	Matthew	Roberts
Eastern Health	Dong	Wang
Fiona Stanley Hospital	Monika	Chang
Fiona Stanley Hospital	Anna	Chiam
Fiona Stanley Hospital	Duncan	Wright
Fiona Stanley Hospital	Orla	O'Brien
Fiona Stanley Hospital	Ramyasuda	Swaminathan
Fiona Stanley Hospital	Samadhi	Wimalasena
Fiona Stanley Hospital	Harish	Puttagunta
Flinders Medical Centre	Jeffrey	Barbara
Flinders Medical Centre	Amanda	Luke
Flinders Medical Centre	Margaret	Pummeroy
Flinders Medical Centre	Kim	Torpey
Gold Coast Hospital and Health Service	Gemma	Nicholls
Gold Coast Hospital and Health Service	Amy	Swinbank
Gold Coast Hospital and Health Service	Thomas	Titus
John Hunter Hospital	Peter	Choi
John Hunter Hospital	Ginger	Chu
John Hunter Hospital	Leanne	Garvey
John Hunter Hospital	Alastair	Gillies

Liverpool Hospital	Josephine	Chow
Liverpool Hospital	Imelda	De Guzman
Liverpool Hospital	Jeanny	Gando
Liverpool Hospital	Jeffrey	Wong
Liverpool Hospital	Richard	Nguyen
Mackay Hospital	Roy	Cherian
Mackay Hospital	Raye	Gillard
Mackay Hospital	Rachel	James
Mater Hospital	Michael	Burke
Mater Hospital	Leanne	Glancy
Mater Hospital	Shimbie	Lewis
Mater Hospital	Richard	Baer
Mater Hospital	Sophie	Wade
Monash Health	Kate	Fitt
Monash Health	Peter	Kerr
Monash Health	Kevan	Polkinghorne
Monash Health	Mechelle	Seneviratne
Nepean Hospital	Muralikrishna	Komala
Nepean Hospital	Junie	McCourt
Nepean Hospital	Craig	Lawlor
Ipswich Hospital	Julia	Bell
Ipswich Hospital	David W	Johnson
Prince of Wales Hospital	Michaela	Kelleher
Prince of Wales Hospital	Sradha	Kotwal
Metro South Hospital and Health Service	Amanda	Coburn
Metro South Hospital and Health Service	Sarah	Guo
Metro South Hospital and Health Service	David W	Johnson
Metro South Hospital and Health Service	Joanna	Sudak
Metro South Hospital and Health Service	Diana	Leary
Rockhampton Hospital	Jenny	Anderson
Rockhampton Hospital	Thin	Han
Rockhampton Hospital	Tresna	Titmarsh
Royal Adelaide Hospital	Emily	Adam
Royal Adelaide Hospital	Bronwyn	Hockley
Royal Adelaide Hospital	Jenny	Latte
Royal Adelaide Hospital	Yvonne	Matthew
Royal Adelaide Hospital	Stephen	McDonald
Royal Adelaide Hospital	Chen Au	Peh
Royal Adelaide Hospital	Rebecca	Taylor
Royal Brisbane Hospital	David	McIntyre
Royal Brisbane Hospital	Sharadchandra	Ratanjee
Royal Darwin Hospital	Karolynn	Maurice
Royal Darwin Hospital	Fiona	Rettie
Royal Darwin Hospital	Madhivanan	Sundaram
Royal Darwin Hospital	Naomi	Grimshaw
Royal Hobart Hospital	Matthew	Jose
Royal Hobart Hospital	Gail	Read
Royal Melbourne Hospital	Jayne	Amy
Royal Melbourne Hospital	Patricia	Coutts
Royal Melbourne Hospital	Maria	Presno
Royal Melbourne Hospital	Nigel	Toussaint
Royal North Shore Hospital	Debbie	Knagge
Royal North Shore Hospital	Colleen	Van Senden
Royal North Shore Hospital	Linh	Pham
Royal North Shore Hospital	Muh Geot	Wong
Royal Prince Alfred Hospital	Jane	Nicholson
Royal Prince Alfred Hospital	Paul	Snelling

Sir Charles Gairdner Hospital	Neil	Boudville
Sir Charles Gairdner Hospital	Alison	Farmer
Sir Charles Gairdner Hospital	Ingrid	Holmes
Sir Charles Gairdner Hospital	Victoria	Link
Sir Charles Gairdner Hospital	Vivien	Perreau
Sir Charles Gairdner Hospital	Nicole	Warnecke
St George Hospital	Sunil V	Badve
St George Hospital	Yanella	Martinez-Smith
St George Hospital	Jayson	Catiwa
St Vincent's Hospital	Frank	Ierino
St Vincent's Hospital	Emmet	O'Flaherty
Sunshine Coast Hospital and Health Service	Nicholas	Gray
Sunshine Coast Hospital and Health Service	Gerald	Hilder
Sunshine Coast Hospital and Health Service	Kaylene	Wadd
Sunshine Coast Hospital and Health Service	Andrea	Pollock
Sunshine Coast Hospital and Health Service	Stanley	Searle
Tamworth Hospital	Cheryl	Wertheim
Tamworth Hospital	Stephen	May
Tamworth Hospital	Jill	Telfer
The George Institute for Global Health	Martin	Gallagher
The George Institute for Global Health	Sradha	Kotwal
The George Institute for Global Health	Sarah	Coggan
The George Institute for Global Health	Casey	Yates
The George Institute for Global Health	Kathryn	Higgins
The George Institute for Global Health	Earl	James
The George Institute for Global Health	Alison	Coenen
The George Institute for Global Health	Kris	Rogers
The George Institute for Global Health	Gian Luca	Di Tanna
The George Institute for Global Health	Jayanthi	Mysore
The George Institute for Global Health	Joseph	Alvin Santos
The George Institute for Global Health	Benjamin	Talbot
The George Institute for Global Health	Katrina	Thistlethwaite
Toowoomba Hospital	Elizabeth	Coroneos
Toowoomba Hospital	Shannon	Nugent
Toowoomba Hospital	Ian	Fox
Toowoomba Hospital	Sree	Venuthurupalli
Western Health	Jennifer	Connor
Western Health	Ruth	Thachaw
Western Health	Sandra	Crikis
Wollongong Hospital	Pauline	Byrne
Wollongong Hospital	Karumathil	Murali
Wollongong Hospital	Hicham	Cheikh Hassan
Western Sydney Local Health District	Lin	Huang
Western Sydney Local Health District	Romiereeza	Dizon
Western Sydney Local Health District	Deepika	Joshi
Western Sydney Local Health District	Jon	Mendoza
Western Sydney Local Health District	Bishi	Augustine
Western Sydney Local Health District	Disha	Balraj
Western Sydney Local Health District	Vincent	Lee
Western Sydney Local Health District	David	O'Donnell
**Other partnering organizations**		
ANZDATA Registry		
Department of Health and Human Services Victoria – Safer Care Victoria		
Kidney Health Australia		
Kidney Health Australia – Caring for Australasians with Renal Impairment		
Queensland Health		
